# Leveraging various extracellular matrix levels to assess prognosis and sensitivity to immunotherapy in patients with ovarian cancer

**DOI:** 10.3389/fonc.2023.1163695

**Published:** 2023-05-09

**Authors:** Youqun Huang, Xingxing Lei, Lisha Sun, Yu Liu, Jiao Yang

**Affiliations:** ^1^ Department of Nephrology-2, Hospital of Chengdu University of Traditional Chinese Medicine, Chengdu, Sichuan, China; ^2^ Department of Endocrinology, Hospital of Chengdu University of Traditional Chinese Medicine, Chengdu, Sichuan, China; ^3^ Department of Nephrology, South China Hospital, Health Science Center, Shenzhen University, Shenzhen, China

**Keywords:** ovarian cancer, ECMS, immune landscape, prognosis, immunotherapy

## Abstract

**Background:**

Ovarian cancer (OC) is the fifth leading cause of cancer-related deaths among women. Late diagnosis and heterogeneous treatment result in a poor prognosis for patients with OC. Therefore, we aimed to develop new biomarkers to predict accurate prognoses and provide references for individualized treatment strategies.

**Methods:**

We constructed a co-expression network applying the “WGCNA” package and identified the extracellular matrix-associated gene modules. We figured out the best model and generated the extracellular matrix score (ECMS). The ECMS’ ability to predict accurate OC patients’ prognoses and responses to immunotherapy was evaluated.

**Results:**

The ECMS was an independent prognostic factor in the training [hazard ratio (HR) = 3.132 (2.068–4.744), p< 0.001] and testing sets [HR = 5.514 (2.084–14.586), p< 0.001]. The receiver operating characteristic curve (ROC) analysis showed that the AUC values for 1, 3, and 5 years were 0.528, 0.594, and 0.67 for the training set, respectively, and 0.571, 0.635, and 0.684 for the testing set, respectively. It was found that the high ECMS group had shorter overall survival than the low ECMS group [HR = 2 (1.53–2.61), p< 0.001 in the training set; HR = 1.62 (1.06–2.47), p = 0.021 in the testing set; HR = 1.39 (1.05–1.86), p = 0.022 in the training set]. The ROC values of the ECMS model for predicting immune response were 0.566 (training set) and 0.572 (testing set). The response rate to immunotherapy was higher in patients with low ECMS.

**Conclusion:**

We created an ECMS model to predict the prognosis and immunotherapeutic benefits in OC patients and provided references for individualized treatment of OC patients.

## Introduction

In 2022, approximately 19,880 patients in the United States were diagnosed with ovarian cancer (OC) and 12,810 patients died from OC. It is the 11th most prevalent cancer and the fifth leading cause of cancer-related deaths among women (1). Indeed, OC includes a variety of pathological types, and epithelial OC is the most frequent pathological type, accounting for approximately 80% (2). The 5-year overall survival (OS) rate after OC diagnosis is only 47% due to failure to diagnose early, metastasis, relapse, and drug resistance (3). The first-line treatment for OC includes surgery and the administration of platinum drugs combined with paclitaxel, and maintenance therapies include bevacizumab and poly(ADP-ribose) polymerase inhibitors. In addition, the idea that immunotherapy has potential effects on various cancers, including OC, has been demonstrated. Therapeutic targeting of the programmed cell death protein 1 (PD-1) and cytotoxic T-lymphocyte antigen 4 (CTLA-4) is effective in many cancers, which can improve the survival rate (4). Previous studies have constructed several models that could predict chemotherapy’s prognosis and efficacy in OC patients (5–8). However, these models do not consider the role of the extracellular matrix (ECM).

The ECM comprises different macromolecules, including glycoprotein, collagens, and proteoglycans, assembled into a three-dimensional supramolecular network to regulate cell growth, survival, motility, and differentiation (9). In addition, ECM is related to the formation of a tumor microenvironment (TME) and its dysregulation can promote tumor progression (10). Deposition of ECM is related to poor outcomes in multiple tumors. For example, in patients with uroepithelial carcinoma of the bladder, inflammatory cancer-associated fibroblasts were significantly associated with poor outcomes (11). In addition, in a study about pancreatic ductal adenocarcinoma, stromal-derived fibroblast growth factor 10 could activate fibroblast growth factor receptor 2 expressed on cancer cells to induce migration and invasion, which was correlated with poor prognosis (12). Similarly, the matrix remodeling gene expression correlated with poor prognosis in breast cancer (BC) patients (13). Abnormal ECM deposition may reduce the effects of chemotherapy and immunotherapy. In preclinical mouse tumor models, inhibition of collagen crosslinking decreased ECM content and tumor stiffness, thereby increasing the efficacy of PD-1 blockade treatment (14). In addition, inhibition of ECM deposition could inhibit colorectal cancer metastasis and enhance the effects of bevacizumab (15). On the contrary, an analysis of pancreatic cancer confirmed that the TGF-b signaling pathway could induce ECM deposition, resulting in the inability to block PD-1 (16). Since ECM is linked to the efficacy and prognosis of many tumor patients, exploring ECM-based prognostic and efficacy prediction models for OC may help the prognostic assessment and individualized treatment strategies to benefit more OC patients.

In this study, we constructed a co-expression network applying the “WGCNA” package and identified the extracellular matrix-associated gene modules. Independent prognostic factors in candidate ECM genes were then screened. We determined the best model utilizing the Cox proportional hazard model with the LASSO penalty. Therefore, a new ECM score (ECMS) model was developed, and its ability to predict accurate OC patients’ prognoses and responses to immunotherapy was evaluated.

## Methods

### Data extraction and data processing

The transcriptome RNA-seq data and the corresponding information of OC patients were downloaded from the Cancer Genome Atlas (TCGA) database (https://cancergenome.nih.gov/) by the Genomic Data Commons platform. We obtained 349 OV samples after excluding participants with lost visits and missing information. We standardized the original fragments per kilobase per million (FPKM) expression data to transcripts per kilobase per million (TPM) and which served as a training set. In addition, from the University of California Santa Cruz (UCSC) Xena platform (https://xena.ucsc.edu/), we downloaded transcriptome RNA-seq data and the corresponding information of 111 OC patients in ICGC database and used them as a testing set. We collected publicly available immunotherapy cohorts to predict immunotherapy response and used them as a validation set. Finally, the IMvigor210 dataset was collected from http://research-pub.gene.com/IMvigor210CoreBiologie. The IMvigor210 cohort contained 298 urothelial carcinoma patients receiving anti-PD-L1 therapies. A total of 1,028 ECM genes were collected from the hallmark dataset on the MSIDGB website (https://www.gsea-msigdb.org/gsea/msigdb/). We included eligible OC samples based on the following criteria: (a) primary diagnosis of ovarian cancer; (b) having a complete gene expression matrix; (c) having well-established clinical follow-up information (including prognosis, stage, and age).

### Screening of candidate ECM genes

The tumor purity and immune activity were assessed by the ESTIMATE algorithm. Then, we built a co-expression network based on transcriptomic data and ESTIMATE results by the “WGCNA” package and identified ECM-associated gene modules. Parameter settings: unsigned network architecture was adopted with a minimum module gene of 30, deepSplit = 2, cutNet = 0.02, and a correlation threshold of 0.9 used to identify genes of the same module. The intersection of the most relevant ECM-associated gene modules with ECM genes was considered candidate ECM genes.

### Construction of the ECMS model

We determined the independent prognostic factors in candidate ECM genes by univariate COX regression. We selected the best predictive model applying the Cox proportional hazard model with the LASSO penalty and set fivefold cross-validation to prevent overfitting. To achieve cross-validated random sampling, we carried out 500 iterations to figure out the most robust model. After 500 iterations, the model with the highest frequency was regarded as the final model and generated the ECMS:


$$ECMS=\sum iCoefficient(mRNA_{i})\times Expression(mRNA_{i})$$

We calculated the concordance index (C-index) utilizing the R package “survcomp.”

Then, we calculated the ECMS of all patients and divided them into the high and low ECMS groups (also called high- and low-risk groups) according to the median ECMS. To assess the model’s prognostic utility, Kaplan–Meier (KM) curves, time-dependent receiver operating characteristic curves (tROC), and univariate and multivariate Cox regression analyses were applied.

### Functional enrichment and immune infiltration analyses

We carried out a single-sample gene set enrichment analysis (ssGSEA) by applying the R package “gsva” based on the molecular markers mentioned in previous studies (17–20). The detailed molecular markers are provided in **Table S1**. In addition, we applied the GSEA to compare two ECMS groups and used the p< 0.05 criterion to discover the significant KEGG pathway. The R package “limma” had been proposed to identify differentially expressed genes (DEGs) between two ECMS groups at a significance threshold of fdr<0.05, FC >2. In addition, we applied the Metascape (http://www.metascape.org) database to carry out functional enrichment analysis. The evaluation of the immune cell infiltration was performed through the R package “CIBERSORT” (21). Applying the ESTIMATE algorithm, we evaluated the tumor purity and immune activity (22). Finally, we collected SNV neoantigens and indel neoantigens samples from Thorsson et al. (23).

### Prediction of immunotherapy response

We calculated the patients’ immunophenoscore (IPS) based on the genetic characteristics of different immune cell phenotypes. A higher IPS indicates a more active immune response and a higher response to immunotherapy. We applied the TIDE algorithm to simulate the mechanism of tumor immune escape to predict the therapeutic effect of patients for immune checkpoint blockers. Finally, we tested the predictive effectiveness of ECMS through the Imvigor210 cohort.

### Cell lines

The OC cell lines A2780 and SKOV3 and the normal ovarian epithelial cell line IOSE-80 were purchased from iCell Bioscience Inc. All the cells were cultured in DMEM with 10% FBS (Biological, Israel).

### RT-qPCR

RNA was extracted using the RNeasy Mini Kit (QIAGEN). The HiScript II Q RT SuperMix for qPCR Kit (Vazyme, China) was used for reverse transcription. ChamQ Universal SYBR qPCR Master Mix (Vazyme, China) was used for RT-qPCR. GAPDH was the housekeeper gene. The results were calculated using the 2-ΔΔCT method.

### Statistical analysis

We utilized R software (version 4.04) to conduct all statistical analyses and graphs. The Wilcoxon test was utilized to measure the differences between the two ECMS groups. Moreover, the chi-square test was applied to compare the differences in proportions. We used a KM plotter to generate survival curves and assessed the differences by log-rank test. We applied the R package “survivalROC” to plot tROC and evaluated the predictive power utilizing the area under the curve (AUC). We applied the R package “survival” to conduct the univariate and multivariate Cox regression analyses and “rms” to plot the nomogram and calibration curves. All tests were two-tailed, and p< 0.05 was considered statistically significant if not otherwise stated.

## Results

### Identification of the candidate ECM genes

**Figure 1:**
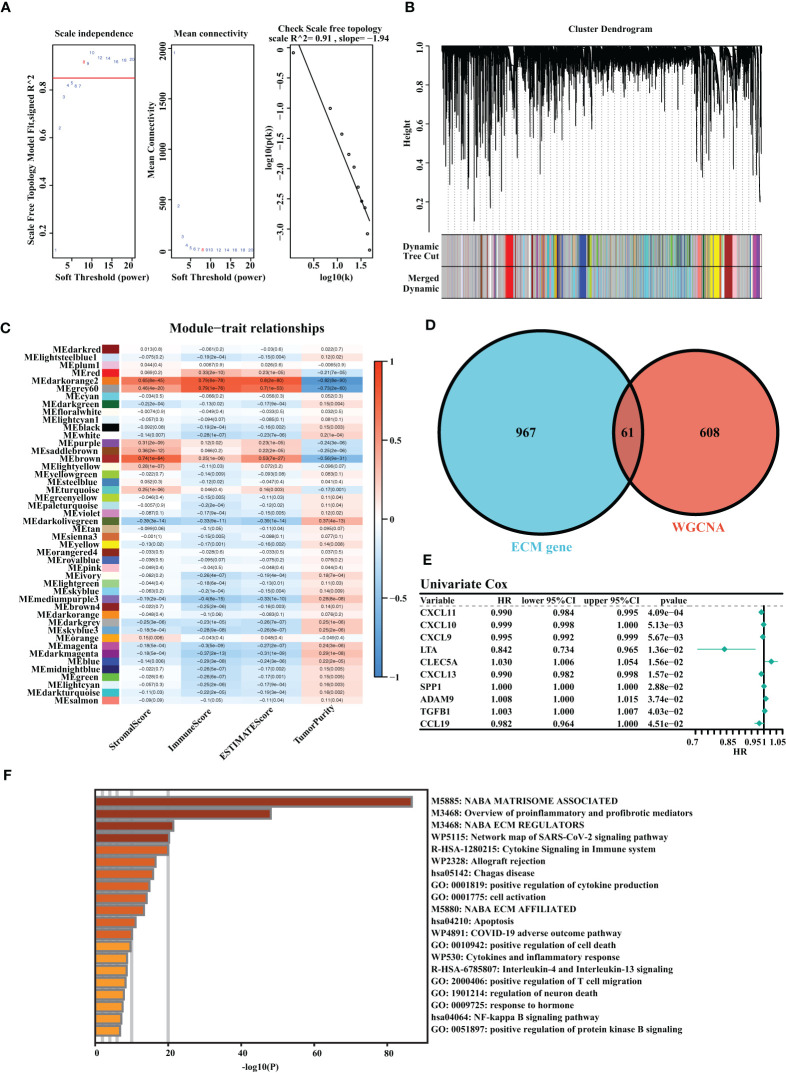
Identification of the highly valuable ECM-related genes. **(A)** The relation between the scale-free topology fit index and soft threshold. **(B)** Gene modules identified by cluster dendrogram. **(C)** Correlation analysis of modules with StromalScore, ImmuneScore, ESTIMATEScore, and TumorPurity. **(D)** Venn diagram of the 1,028 ECM genes and 661 ECM-associated genes from WGCNA. **(E)** Forest plots presenting univariate Cox regression analyses of candidate ECM genes as independent prognostic factors. **(F)** Enrichment analysis of the candidate ECM genes.

A total of three OC cohorts (TCGA-OV, ICGC-OV, and Imvigor210) were considered suitable for this study. We collected 1,028 ECM genes from the hallmark dataset on the MSIDGB website. The WGCNA algorithm was applied to determine ECM-associated genes. The scale-free network was constructed with the scale-free topology fitting index set to 0.9, and the corresponding optimal soft threshold value was 8 (**Figure 1A**). We used a clustering dendrogram to identify 46 modules (**Figure 1B**). The correlation coefficient between the Darkorange2 module and ImmuneScore was 0.79, and the correlation coefficient between the Darkorange2 module and ESTIMATEScore was 0.8, suggesting that the Darkorange2 module was selectively expressed in samples with high immune cell infiltration (**Figure 1C**). The 1,028 ECM genes and 669 genes from the most relevant gene modules were intersected to obtain 61 candidate ECM genes (**Figure 1D**). These 61 candidate ECM genes were screened for independent prognostic factors by doing univariate Cox regression analysis, and we identified 10 genes (**Figure 1E**). To comprehensively analyze these genes, we used Metascape for functional enrichment analysis. We listed the top 20 enrichment terms in which candidate ECM genes were mostly enriched in NABA MATRISOME-ASSOCIATED signaling pathways (**Figure 1F**).

### Construction and validation of the ECMS model

**Figure 2:**
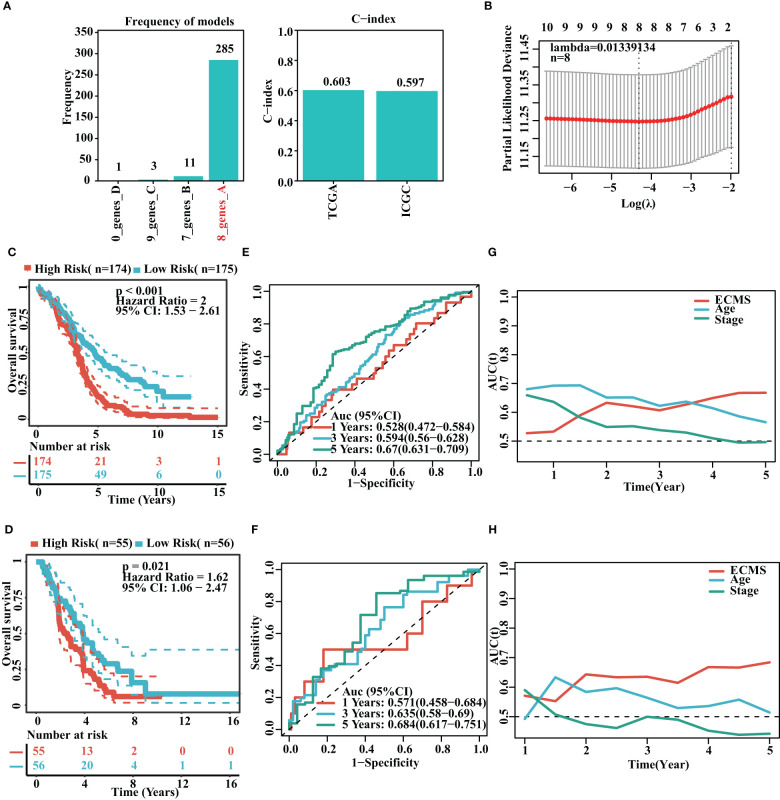
Construction of risk model. **(A)** Frequency of models and C-indices of training and testing sets. **(B)** The selection of the tuning parameter (λ) in the LASSO model. **(C, D)** Survival curves of the training **(C)** and testing sets **(D)**. **(E, F)** ROC curves of ECMS in the training **(E)** and testing sets **(F–H)**. Time-dependent AUC values for the ECMS and clinical characteristics in the training **(G)** and testing sets **(H)**.

It was found that the risk model containing eight genes was the best one (**Figure 2A**). Detailed information on the eight genes is shown in **Table S2**. As we know, C-index is used to assess prediction capacity and reliability. The C-indexes were 0.603 (training set) and 0.597 (testing set). The details are shown in **Figure 2A**. We constructed the risk model containing eight genes based on the optimal λ value of 0.01339134 (**Figure 2B**). The survival analysis demonstrated that the high-risk group had shorter OS than the low-risk group in the training set [hazard ratio (HR) = 2 (1.53–2.61), p< 0.001, **Figure 2C**]. Moreover, the testing set showed similar results [HR = 1.62 (1.06–2.47), p = 0.021, **Figure 2D**]. To further test the validity of the ECMS, we performed ROC analysis on the training and testing sets. We used the AUC analysis to assess the reliability of our signature. The AUC values of 0.528, 0.594, and 0.67 for the training set (**Figure 2E**) at 1, 3, and 5 years, respectively, and 0.571, 0.635, and 0.684 for the testing set (**Figure 2F**), respectively. The tROC analysis indicated that ECMS was a reliable predictor for OC patients (**Figures 2G, H**).

**Figure 3:**
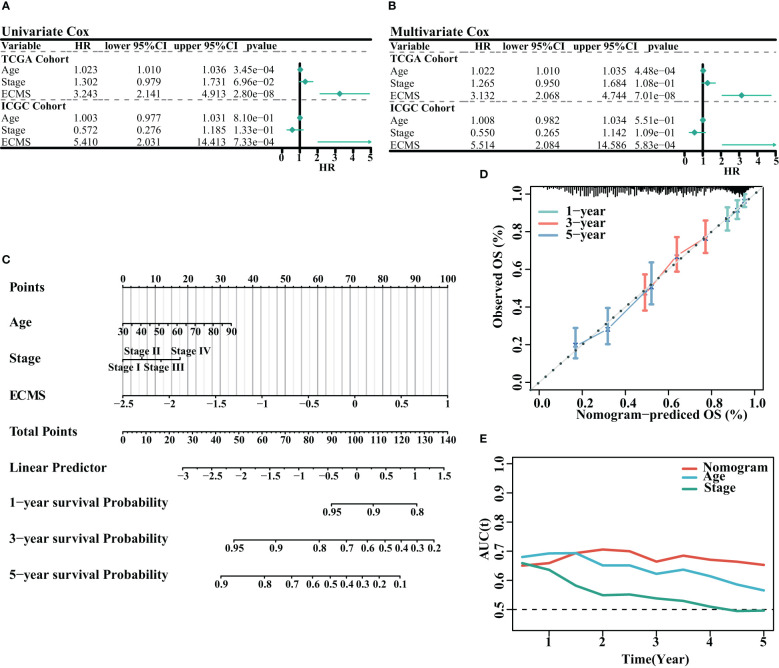
Assessment of the prognostic value of ECMS. **(A, B)** Forest plots of univariate regression analyses **(A)** and multivariable regression analyses **(B)** involving the ECMS, age, and stage. **(C)** Nomogram showing the risk assessment of patients. **(D)** Nomogram calibration curves predicting the OS at 1, 3, and 5 years. **(E)** Time-dependent AUC values of nomogram, age, and stage.

We analyzed the association between age, stage, ECMS, and prognosis. The ECMS was an independent risk factor according to the univariate Cox regression analysis [HR = 3.243 (2.141–4.913), p< 0.001 in the training set; HR = 5.410 (2.031–14.413), p< 0.001 in the testing set, **Figure 3A**]. In multivariate Cox regression analysis, ECMS also exhibited an excellent prognostic performance [HR = 3.132 (2.068–4.744), p< 0.001 in the training set; HR = 5.514 (2.084–14.586), p< 0.001 in the testing set, **Figure 3B**]. We constructed a nomogram to assess the survival probability for OC patients (**Figure 3C**). The calibration curve analysis indicated this nomogram was accurate (**Figure 3D**). In addition, the tROC analysis revealed that the nomogram outperformed other variables (**Figure 3E**).

### Enrichment analysis

**Figure 4:**
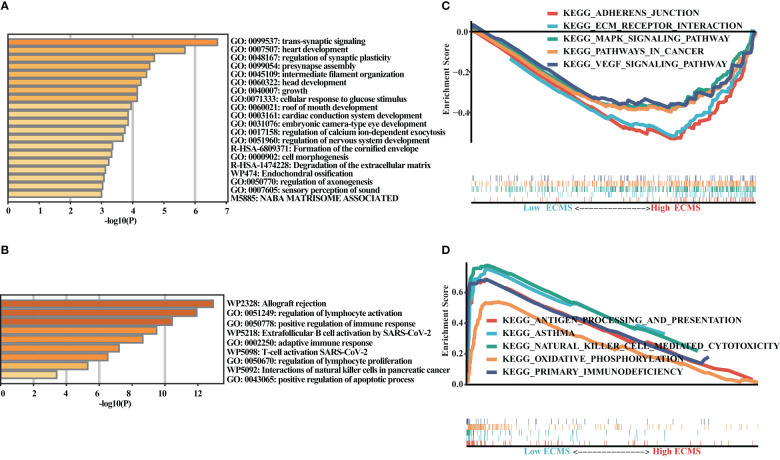
Functional enrichment analysis. **(A, B)** Enrichment analysis of the DEGs in the high **(A)** and low **(B)** ECMS groups; **(C, D)** Enrichment plots of GSEA in the high **(C)** and low **(D)** ECMS group.

We obtained DEGs and input these genes into Metascape. It was observed that the genes elevated in the high ECMS group were significantly related to trans-synaptic signaling, heart development, regulation of synaptic plasticity, presynapse assembly, and intermediate filament organization (**Figure 4A**). Moreover, the genes elevated in the low ECMS group were notably connected with allograft rejection, regulation of lymphocyte activation, positive regulation of immune response, extrafollicular B-cell activation by SARS-CoV-2, and adaptive immune response (**Figure 4B**). In addition, functional enrichment analyses in the high ECMS group showed that adherens junction, ECM receptor interactions, mitogen-activated protein kinase (MAPK) signaling pathway, pathways in cancer, and vascular endothelial growth factor (VEGF) signaling pathway were enriched (**Figure 4C**). In contrast, in the low ECMS group, antigen processing and presentation, asthma, natural killer cell-mediated cytotoxicity, oxidative phosphorylation, and primary immunodeficiency were mainly enriched (**Figure 4D**). We also performed an enrichment analysis on the validation set. The results revealed that elevated genes were mainly associated with spliceosome in the high ECMS group (**Figure S1A**) and were mainly related to inflammatory response, neutrophil degranulation, positive regulation of cytokine production, phagosome, and osteoclast differentiation in the low ECMS group (**Figure S1B**). The GSEA revealed that in the high ECMS group, adherens junction, gap junction, MAPK signaling pathway, o-glycan biosynthesis, and pathways in cancer were mostly enriched (**Figure S1C**), whereas oxidative phosphorylation, primary immunodeficiency, protein export, and ribosome were enriched in the low ECMS group (**Figure S1D**).

### Immune landscape

**Figure 5:**
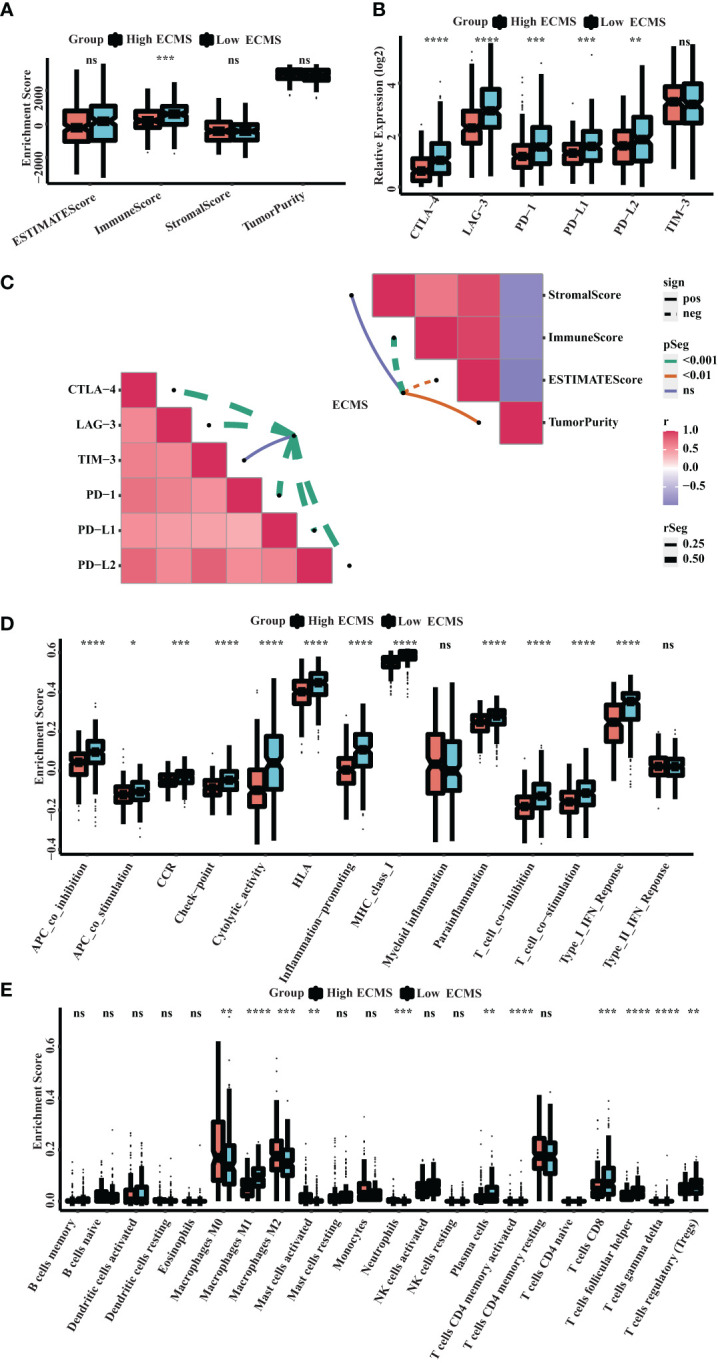
Immune landscape in the high and low ECMS group. **(A)** Box plot of the enrichment score. **(B)** Box plot of the immune checkpoints expression. **(C)** Heatmap of the correlation between the ECMS and enrichment score, as well as immune checkpoint expression, respectively. **(D)** Box plots of the immune pathway activity. **(E)** Box plots of the proportion of immune cell infiltration. *p< 0.05; **p< 0.01; ***p< 0.001; ****p< 0.0001; ns, no significance.

ssGSEA showed a significant difference in ImmuneScore between the two ECMS groups, with the low ECMS group exhibiting higher immune activity (**Figure 5A**). In addition, we selected CTLA-4, T-cell immunoglobulin and mucin domain 3 (TIM-3), PD-1, PD-L1, PD-L2, and lymphocyte-activation gene 3 (LAG3) as biomarkers of immune checkpoint activity. We analyzed the differences of their expression between two ECMS groups. We found that the expression was notably more active in the low ECMS group (**Figure 5B**). Then, we analyzed the correlation between ECMS and enrichment scores and the relationship between ECMS and differences in immune checkpoint expression, as shown in **Figure 5C**. Subsequently, we assessed immune cell infiltration fraction and pathway activity in two groups. The activity of most immune pathways was notably lower in the high ECMS group (**Figure 5D**). A significant difference was seen between the two ECMS groups in the infiltration degree of most immune cells (e.g., T cells, macrophages, mast cells), as shown in **Figure 5E**. We also performed ssGSEA on the validation set to assess the immune-related pathways’ activity. The enrichment score between both groups was not significantly different (**Figure S2A**), but there were differences in the expression of LAG3 and TIM-3 (**Figure S2B**). The heat map revealed the correlation between ECMS and enrichment score and the expression difference of immune checkpoint (**Figure S2C**).

### Prediction of immunotherapy response

**Figure 6:**
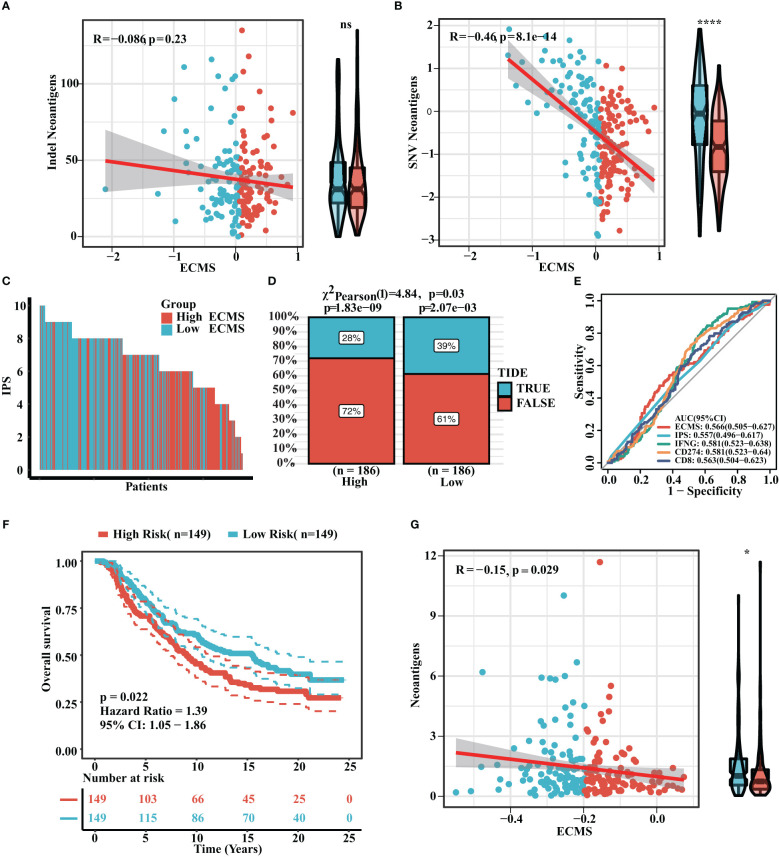
Prediction of immunotherapy response. **(A)** Correlation between indel neoantigens and ECMS. **(B)** Correlation between SNV neoantigens and ECMS. **(C)** IPS of the patients in TCGA cohort. **(D)** Distribution of the response rate to anti-PD-L1 immunotherapy in the high- and low-ECMS group. **(E)** ROC curves of ECMS, IPS, IFNG, CD274, and CD8 in TCGA cohort. **(F)** Survival curves of the Imvigor210 cohort. **(G)** Correlation between the neoantigens and ECMS. *p< 0.05; ****p< 0.0001; ns, no significance.

Neoantigen is one of the biomarkers of immunotherapy, which can guide the application of immunotherapy. We analyzed the correlation between indel neoantigens, SNV neoantigens, neoantigens, and ECMS, and the results are presented in **Figures 6A, B**. There were significant negative correlations between SNV neoantigens and ECMS (R = -0.46, p< 0.0001, **Figure 6B**), whereas no correlation was observed between indel neoantigens and ECMS (p = 0.23, **Figure 6A**). IPS can be used to assess the response to immunotherapy. The IPS of patients in TCGA and ICGC cohorts are shown in **Figure 6C** and **Figure S3A**. In addition, the response rate to anti-PD-L1 immunotherapy in the training set was higher in the low ECMS group (p = 0.03) (**Figure 6D**). The testing set presented similar results (p = 0.01) (**Figure S3B**). Then, we performed a ROC analysis on TCGA set, and the AUC value was 0.566 (**Figure 6E**). In contrast, the AUC value in the IGCA set was 0.572 (**Figure S3C**), indicating that ECMS was a more reliable predictor than other commonly used indicators. We also found that the high ECMS group had shorter OS than the low ECMS group in the Imvigor210 cohort (hazard ratio = 1.39, p = 0.022, **Figure 6F**). Moreover, we also found a significant negative correlation between neoantigens and ECMS (**Figure 6G**).

### Verify the expression of ECMS genes in ovarian cancer cell lines

**Figure 7:**
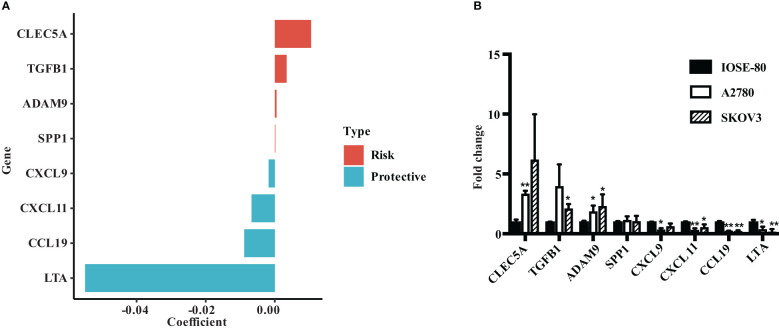
Verify the expression of ECMS genes in ovarian cancer cell lines. **(A)** The coefficient index of genes in the ECMS model. **(B)** The expression levels of ECMS genes in OC and ovarian epithelial cell lines detected by qRT-PCR. *p < 0.05; **p < 0.01.

We evaluated the risk coefficients of genes in the ECMS model. Among them, CLEC5A is the strongest risk factor whereas LTA is the strongest protective factor (**Figure 7A**). Then, we performed RT-qPCR to verify our result (**Figure 7B**). *CLEC5A*, *ADAM9*, and *TGFB1* were highly expressed in OC cell lines compared with the normal ovarian epithelial cell line, whereas *LTA*, *CCL19*, *CXCL11*, and *CXCL9* were downregulated in OC cell lines. However, the expression level of SPP1 showed no difference between normal and malignant ovarian epithelial cell lines (**Figure 7B**).

## Discussion

In the present research, we explored the role of ECM in OC patients and linked them for the first time with the prognosis and effectiveness of immunotherapy. Our results suggested that the ECMS model performed well. The AUC values for the training set at 1, 3, and 5 years were 0.528, 0.594, and 0.67, respectively. We also found that the high ECMS group had shorter OS than the low ECMS group. In addition, the immune landscape demonstrated that the immune checkpoints’ expression was more active in the low ECMS group, and the response rate to anti-PD-L1 treatment was lower in the high ECMS group. The ROC values of the ECMS model for predicting immune response were 0.566 (validation set) and 0.572 (testing set), indicating that the model could predict, to some extent, the response rate to immunotherapy.

The ECM plays a role in regulating cell growth, motility, and differentiation (6). The most widely known ECM alteration in tumor tissue is increased collagen deposition. The increased collagen deposition affects the properties of the TME, thereby modulating cancer cell polarity, migration, and signaling transduction (24–27). Previous studies have shown that increased expression of proteins mediating ECM remodeling can increase mortality in patients with BC, lung cancer, or gastric cancer (GC) (28, 29). In addition, histological studies have observed excessive ECM deposition and remodeling in OC. The fibrosis rich in COL6/collagen VI and fibronectin is already present around the micro metastases, which develops into an extensive connective tissue proliferative TME as the disease progress. COL6 is involved in tumor growth and apoptosis escape in early metastases of OC (30). These findings confirm that ECM is closely associated with the clinical manifestations and prognosis of OC.

It was observed that candidate ECM genes were mostly enriched in the NABA MATRISOME-ASSOCIATED signaling pathway, which had been mentioned to be associated with tumor development (31). The GSEA was performed, and the results indicated that the high ECMS was enriched in adherens junction, ECM receptor interactions, MAPK signaling pathway, pathway in cancer, and VEGF signaling pathway. Adherens junction is cell–cell adhesion complexes that take part in embryogenesis and tissue homeostasis (32). ECM receptor interactions regulate cell behavior and are vital in cell proliferation, adhesion, and migration (33). The MAPK signaling pathway regulates various cellular processes, such as cell proliferation and differentiation (34). The VEGF signaling pathway is a major regulator of angiogenesis and vascular permeability (35). These pathways are involved in tumorigenesis, progression, invasion, and metastasis (36–39). The results indicated that the tumors were developing and metastasizing. In the low ECMS group, enrichment of immune-related pathways such as antigen processing and presentation, asthma, oxidative phosphorylation, natural killer cell-mediated cytotoxicity, and primary immunodeficiency were observed. This suggested that low ECMS patients presented powerful immune function.

We also found that the high ECMS group had shorter OS than the low ECMS group. It could be explained by the enrichment of tumor-related signaling pathways and active tumor growth in the high ECMS group. On the other hand, the low ECMS group was enriched in antigen processing and signal presentation pathways, which showed stronger immune function, thus helping the body to clear tumor cells. Furthermore, the ECMS model was based on candidate ECM genes mainly enriched in NABA MATRISOME-ASSOCIATED signaling pathways associated with tumor development. Thus, this could also explain why the high ECMS group had shorter OS time. Similar results were also seen in the study by Liu et al., who classified BC patients into two groups based on the ECM index (ECMI), which was based on ECM-associated immunogens, and assessed their clinical, biological, and genomic characteristics. The researchers believed the low ECMI group had significantly improved OS (40). In addition, Yang et al. used a gene set variation analysis algorithm to establish ECM scores, and higher ECM scores predicted poor prognosis in GC (41). Similarly, Ding et al. established a new immune-related signature to stratify the risk of OC patients and then predict the prognosis (7). Considering the role of ECM in OC, our study constructed a risk model on the basis of ECM, and the results suggested that ECMS can well predict the prognosis of OC patients.

Tumor-associated ECM may have immunomodulatory effects, influencing antitumor immunity by controlling the localization and migration of immune cells (42). Thus, ECM may influence the effect of immunotherapy. Indeed, previous studies have proved that combination therapy targeting the immune and stromal microenvironment had better therapeutic effects (43, 44). Therefore, we developed an ECMS model to predict the patients’ responses to immunotherapy. The high ECMS group was observed to have lower immune pathway activity. This indicated that ECM might affect the immune regulation of OC. Then, we selected CTLA-4, LAG3, PD-1, PD-L1, PD-L2, and TIM-3 as markers of the immune checkpoint. A significant difference was seen between the two ECMS groups, with more active expression in the low ECMS group. We also found that the response rate to anti-PD-L1 immunotherapy was lower in the high ECMS group in both the validation and testing sets. This could be due to the higher immune pathway activity and more active expression of immune checkpoints in the low ECMS group, so it has a higher response rate to the immune checkpoint inhibitors. Similarly, Mao et al. established a stromal score and investigated the relationship between immunotherapy-related markers or immune cell types and the stromal score in GC. The results of this study also confirmed that the stroma was related to immunotherapy-related markers (45). This is the first study that proposes the role of ECM in predicting immunotherapeutic response in OC. Our study confirmed the association between ECM and the immune-related pathway of OC and the immunotherapeutic response. Improving the OS of OC patients is a common problem in advanced OC. Precision therapy is a good entry point. Our study revealed that the low ECMS group had higher immune pathway activity, the more active expression of the immune checkpoint, and a higher response rate to immune checkpoint inhibitors. Therefore, we can identify OC patients who may benefit from immunotherapy based on the established ECMS model and help clinicians and patients to make individualized treatment decisions.

Nevertheless, this research has several limitations. First, our data are from TCGA, ICGC, and publicly available immunotherapy cohorts, which need to be verified with large samples in reality. Second, the immunotherapy cohort is an advanced uroepithelial cancer cohort with PD-L1 immunotherapy (Imvigor210), and further validation in an OC immunotherapy cohort is needed in the future. Third, the ECMS model needs to be authenticated in reality before application.

In conclusion, we created an ECMS model to predict the prognosis and immunotherapeutic benefits in OC patients and provided references for individualized treatment of OC patients.

## Data availability statement

The original contributions presented in the study are included in the article/**Supplementary Material**. Further inquiries can be directed to the corresponding author.

## Author contributions

YH, LS: designed the study, analyzed the data and wrote the manuscript. YL: established the methods. JY: reviewed and revised the manuscript. All authors contributed to the article and approved the submitted version.
