# Supplementary material for: Leveraging various extracellular matrix levels to assess prognosis and sensitivity to immunotherapy in patients with ovarian cancer
Source: Front Oncol. 2023 May 9;13:1163695. doi: 10.3389/fonc.2023.1163695 (PMC10203472; doi:10.3389/fonc.2023.1163695)
Supplement: Supplementary file 2 [file Table_2.doc]

**Table S2. Detailed information of the 8 ECMS genes.**

| **Gene** | **Full name** |
| --- | --- |
| CLEC5A | C-Type Lectin Domain Containing 5A |
| TGFB1 | Transforming Growth Factor Beta 1 |
| ADAM9 | ADAM Metallopeptidase Domain 9 |
| SPP1 | Secreted Phosphoprotein 1 |
| CXCL9 | C-X-C Motif Chemokine Ligand 9 |
| CXCL11 | C-X-C Motif Chemokine Ligand 11 |
| CCL19 | C-C Motif Chemokine Ligand 19 |
| LTA | Lymphotoxin Alpha |
