# Supplementary figures and images for: Leveraging various extracellular matrix levels to assess prognosis and sensitivity to immunotherapy in patients with ovarian cancer
Source: Front Oncol. 2023 May 9;13:1163695. doi: 10.3389/fonc.2023.1163695 (PMC10203472; doi:10.3389/fonc.2023.1163695)

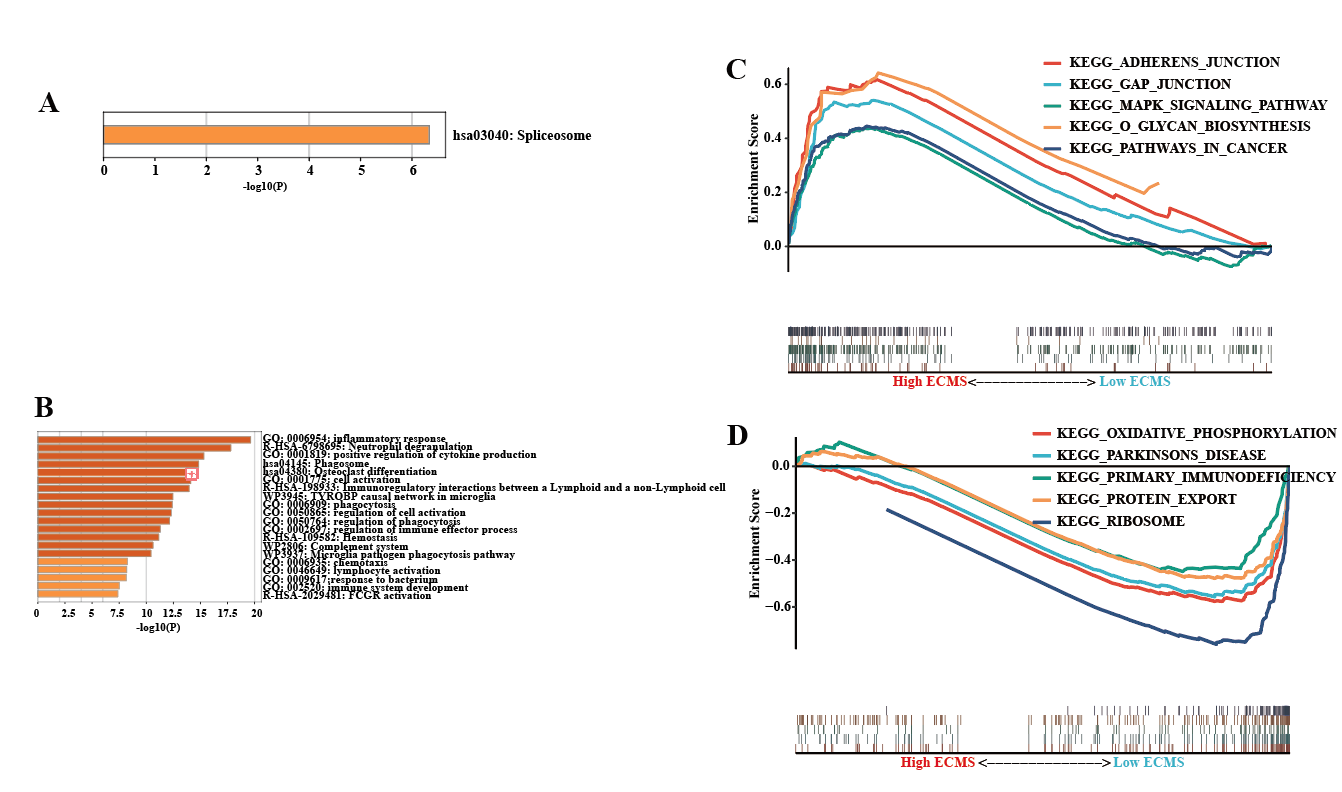

Supplement: Supplementary file 3 [file Image_1.tif]

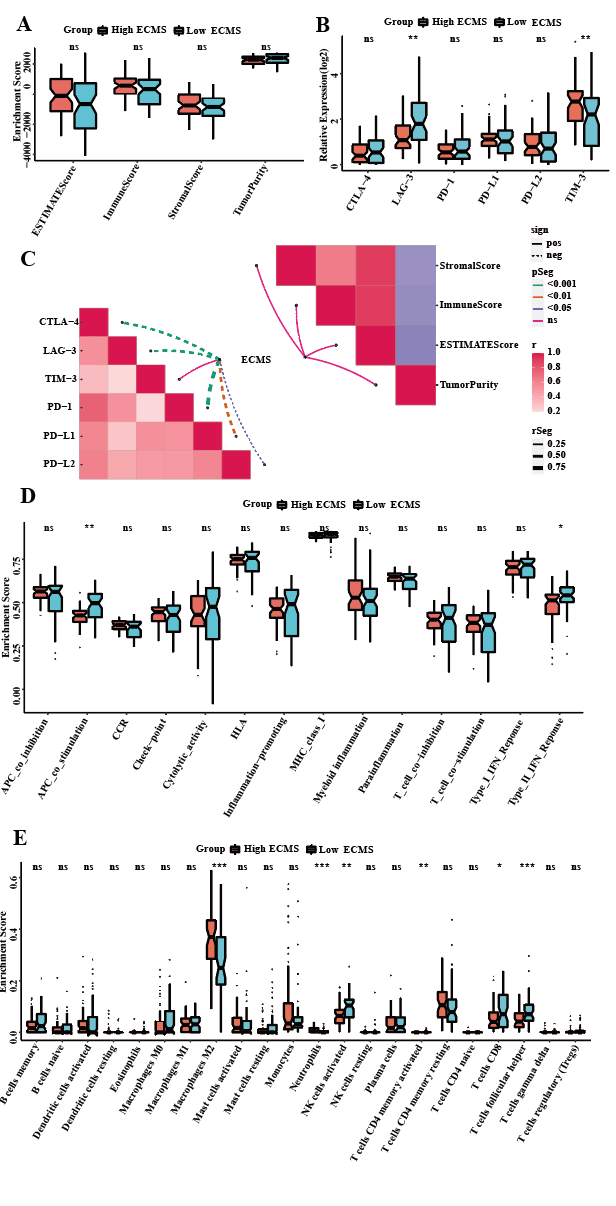

Supplement: Supplementary file 4 [file Image_2.tif]

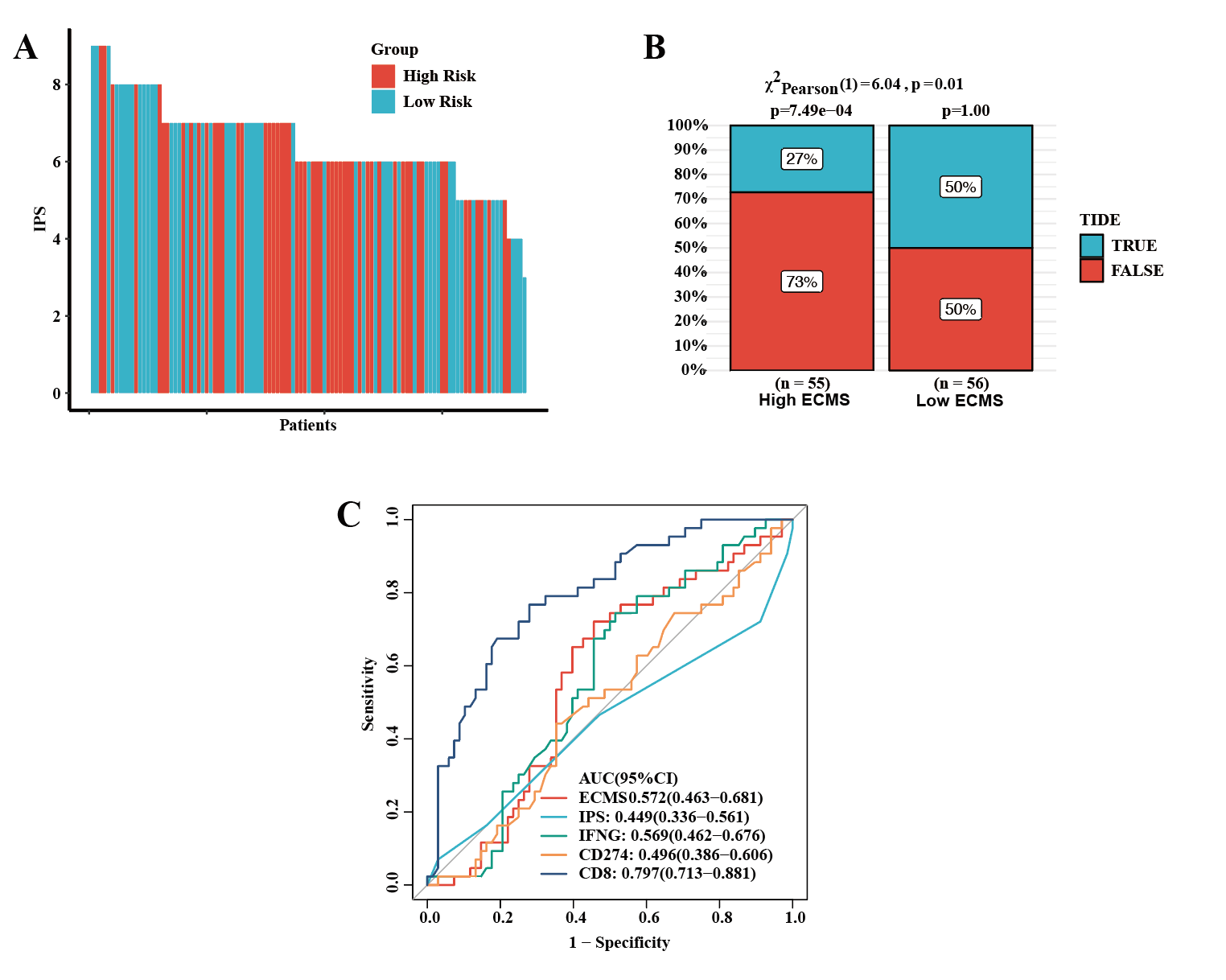

Supplement: Supplementary file 5 [file Image_3.tif]
